# Exploring biomarkers for noise-induced hearing loss through mitochondrial DNA methylation analysis

**DOI:** 10.3389/fphar.2025.1561791

**Published:** 2025-07-04

**Authors:** Dianpeng Wang, Caiping Li, Liuwei Shi, Dafeng Lin, Shaofan Weng, Xiangli Yang, Peimao Li, Zhimin Zhang, Wen Zhang, Yan Guo, Guangtao Yang, Zhenlie Huang, Naixing Zhang

**Affiliations:** ^1^ School of Public Health, Southern Medical University, Guangzhou, China; ^2^ Shenzhen Prevention and Treatment Center for Occupational Diseases, Shenzhen, China; ^3^ School of Public Health, Jilin University, Changchun, China

**Keywords:** noise-induced hearing loss, mitochondrial DNA, methylation, Atp6, CYB

## Abstract

**Objective:**

Noise-induced hearing loss (NIHL), resulting from occupational noise exposure, is a significant health concern with considerable economic and social implications. It is the most commonly reported occupational disease in developing countries. Noise causes cochlear cell damage by inducing mitochondrial oxidative stress elevating reactive oxygen species (ROS), ultimately leading to cell apoptosis. This study explores the impact of noise-induced oxidative stress on mitochondrial DNA methylation and aims to identify potential molecular biomarkers for NIHL.

**Methods:**

This study included 40 cases of NIHL and 40 controls. Mitochondrial genome-wide methylation sequencing was performed using a targeted region approach with bisulfite multiplex PCR capture technology and high-depth next-generation sequencing (NGS).

**Results:**

The analysis revealed significant differences in methylation levels at 53 sites within mitochondrial genes, including 12S_rRNA, 16S_rRNA, tRNA-Ile, ND2, tRNA-Trp, CO1, CO2, ATP6, and CYB, with lower methylation levels observed in the case group compared to controls. In contrast, methylation levels at 31 sites, including 12S_rRNA, tRNA-Val, 16S_rRNA, CO1, CO3, ND3, tRNA-Arg, ND4, and ND5, were significantly higher in the case group. Receiver Operating Characteristic (ROC) curve analysis showed that the CYB gene had an area under the curve (AUC) of 0.807, with high sensitivity (0.90) and reasonable specificity (0.70).

**Conclusion:**

This study demonstrates a reduction in mitochondrial DNA methylation, particularly in the ATP6 and CYB genes, among individuals with NIHL. These findings suggest that mitochondrial DNA methylation, especially in the CYB gene, could serve as a potential biomarker for NIHL. However, given the complex interplay of various factors, including genetic, environmental, and lifestyle influences, further research is needed to fully understand the role of mitochondrial DNA methylation and oxidative stress in NIHL. Future studies should focus on identifying additional biomarkers and elucidating their mechanistic relationships, which could lead to more accurate diagnostic tools and therapeutic strategies.

## 1 Introduction

A report from the World Health Organization (WHO) indicates that approximately 16% of the global workforce is impacted by high-level noise, with 7%–21% of occupational hearing loss attributed to noise exposure (Xin et al., 2023). The prevalence of this issue is particularly high in developing countries. Each year, an estimated one million workers experience varying degrees of hearing loss due to excessive noise exposure. Noise-Induced Hearing Loss (NIHL) is a form of sensorineural hearing loss resulting from damage to inner ear hair cells caused by intense noise stimulation (Natarajan et al., 2023). One mechanism underlying noise-induced hearing loss involves the aberrant dilation of blood vessels in the auditory system due to the mechanical impact of sound waves (Wang et al., 2020). This disruption of inner ear microcirculation results in ischemia and hypoxia in inner ear cells, ultimately leading to dysfunction of the cochlea and hair cells (Shu et al., 2019). Ischemia and hypoxia can trigger an elevation in ROS produced by mitochondria, subsequently causing damage to cellular and subcellular components (Tabuchi et al., 2010). Mitochondria serve as key locations for cellular energy production, oxygen metabolism, and the generation of free radicals. Decreased blood flow within the cochlea and cochlear nucleus due to noise stimulation results in inner ear ischemia and hypoxia, leading to a reduction in antioxidant capacity and hindered removal of free radicals (Zhou et al., 2023). Consequently, inner ear oxidative stress is heightened, causing sensitivity and damage to mitochondria post-noise exposure. Epigenetic mechanisms, particularly DNA methylation, play a critical role in the development and advancement of hearing loss (Tisato et al., 2023). Studies have shown that aberrant regulation of DNA methylation, resulting in epigenetic changes, can impact the development of cochlear tissue and contribute to hearing impairment (Zheng et al., 2021). In addition, recent research has linked NIHL to neurodegenerative diseases such as Alzheimer’s disease. For instance, a study using 3xTgAD/Polβ ± AD mice revealed a significant reduction in hair cells and paired ribbon synapses of inner hair cells (IHCs) in the 32 kHz region of the cochlea, alongside downregulation of mitochondrial SIRT3 expression (Park et al., 2024). These findings suggest that mitochondrial epigenetic dysregulation in the cochlea may also contribute to cognitive decline associated with NIHL.Therefore, this research investigates the potential regulatory function of mtDNA methylation in the pathogenesis of noise-induced hearing loss, considering the involvement of mitochondria in this condition and the influence of methylation on cochlear cell metabolism.

The body of research concerning alterations in methylation levels among individuals with occupational noise-induced hearing loss is notably sparse, particularly with respect to studies concentrating on mitochondrial gene methylation. This investigation aimed to assess the methylation levels across the entire mitochondrial genome and to determine mitochondrial copy number in a cohort of 80 subjects. Furthermore, the study evaluated antioxidant markers, including superoxide dismutase (SOD), malondialdehyde (MDA), and total antioxidant status (TAS). This study presents a TBS detection method for mitochondrial whole-genome methylation, which is anticipated to enhance the understanding of the pathogenic mechanisms underlying oxidative damage in individuals with noise-induced hearing loss or noise-induced hearing impairment.

## 2 Materials and methods

### 2.1 Research object

The study focuses on cases who have undergone physical examinations at the Shenzhen Prevention and Treatment Center for Occupational Diseases, specifically diagnosed patients with noise-induced hearing loss. The inclusion criteria for the case group, consisting of 40 patients, are determined by the guidelines outlined in GBZ49-2014 “Diagnosis of Occupational Noise-Induced Hearing Loss,” including a history of occupational noise exposure of at least 3 years and an average hearing threshold of both ears at high frequencies (3000Hz, 4000Hz, 6000 Hz) of 40 dB or higher. Exclusion criteria encompass conditions such as pseudohypoacusis, exaggerated hearing loss, drug-induced hearing impairment, traumatic hearing loss, infectious hearing loss, genetic hearing loss, Ménière’s disease, sudden deafness, acoustic neuroma, and auditory nerve disease.

The control group comprises 40 healthy individuals who have not been exposed to noisy working environments. Inclusion criteria involve ensuring an age difference of no more than 5 years. Additionally, participants must have a pure tone audiometry result showing an average high-frequency threshold of less than 35 dB and a threshold of 25 dB or lower for any frequency band (500Hz, 1000Hz, 2000 Hz) in either ear. Exclusion criteria encompass a history of head trauma, mumps, measles, rubella, otitis media, Ménière’s syndrome, tympanic membrane perforation, and a history of ototoxic drug use. This study has been approved by the Ethics Committee of Shenzhen Prevention and Treatment Center for Occupational Diseases, and all subjects were informed and provided consent.

### 2.2 Sample collection

Each experimental subject should have 2.0 mL of upper limb venous blood collected and transferred into regular biochemical tubes without anticoagulant and EDTA (Ethylenediaminetetraacetic acid) anticoagulant tubes. It is advisable to divide the blood samples into two tubes during collection to aid in the subsequent DNA extraction from both patients and normal individuals.

### 2.3 Instruments and reagents

#### 2.3.1 Equipment

The specific equipment utilized for this research includes the Speed Regulating Mini Centrifuge (Instrument model: Super Mini Dancer manufactured by Shanghai Bioengineering Co, Ltd.), and the Fluorescence Quantitative PCR (Polymerase Chain Reaction) Instrument (Instrument model: StepOnePlus manufactured by Applied Biology Co., Ltd. in the United States). The reagent employed in this study is the SuperReal Color Fluorescence Quantitative Premixed Reagent (NO. FP215) from Tiangen Biochemical Technology. Superoxide dismutase assay kit (substrate method), total antioxidant status assay kit (colorimetric method), and glutathione peroxidase assay kit (UV enzyme method) were all purchased from Shandong Zhongtuo Biotechnology Co., Ltd.

### 2.4 Methods

#### 2.4.1 The determination of antioxidant biomarkers

The system involves the collection of 2.0 mL of peripheral blood, centrifugation at 2,000 × g for 30 min, and the collection of serum as the testing sample. The analysis of these biomarkers is conducted in accordance with the manufacturer’s instructions and instrument manual. Specifically, for the measurement of superoxide dismutase (SOD), a mixture of 15 μL of sample (calibration) and 225 μL of R1 reagent [Tris buffer (100.0 mmol/L), ethylene glycol-bis(2-aminoethylether)-N,N,N′,N′-tetraacetic acid (EGTA) (1.0 mmol/L)] is incubated at 37°C for 5 min, and the absorbance A1 is then read. Add 75 μL of R2 reagent containing catechol (20.0 mmol/L), bovine serum albumin (1.0 g/L), and sodium azide (0.5 g/L) to the sample, incubate at 37°C for 5 min, measure the absorbance A2, calculate the difference in absorbance ∆ A = A2-A1, sample concentration = sample (∆ A)× standard concentration/standard (∆ A).

Glutathione peroxidase (GPX) activity was measured by mixing 10 μL of serum with 200 μL of R1 reagent containing Tris buffer (100 mmol/L), potassium carbonate (66.58 g/L), glutathione (4 mmol/L), glutathione reductase (0.5 kU/L), reduced form of coenzyme Ⅱ (NADPH) (0.18 mmol/L), and ethylenediaminetetraacetic acid (EDTA) (0.5 mmol/L). The mixture was incubated at 37°C for 5 min and the absorbance A was read. Subsequently, 50 μL of R2 reagent containing Tris buffer (100 mmol/L), potassium carbonate (66.58 g/L), and hydrogen peroxide isopropylbenzene (0.18 mmol/L) was added. Mix and monitor continuously for 2 min, calculate ∆A/min, ∆A = absorbance at the end of reaction - absorbance at the beginning of reaction. Sample concentration = sample (∆ A/min) × standard concentration/standard (∆ A/min).

Total antioxidant status (TAS): To determine the Total Antioxidant Status (TAS), a serum sample of 10 μL is combined with 200 μL of R1 reagent containing Tris buffer (100 mmol/L), potassium carbonate (66.58 g/L), and ethylenediaminetetraacetic acid (EDTA) (0.5 mmol/L). The mixture is then incubated at 37°C for 5 min and the absorbance A1 is measured. Subsequently, 50 μL of R2 reagent containing Tris buffer (100 mmol/L), potassium carbonate (66.58 g/L), and 2,2-azinobis (3-ethylbenzothiazoline-6-sulfonic acid) diammonium salt (ABTS) (0.61 mmol/L) is added to the mixture. Mix and delay for 5 min, read the absorbance A2, and calculate ∆A, ∆A = absorbance at the end of reaction - absorbance at the beginning of reaction. Sample concentration = sample (∆A)×standard concentration/standard (∆A).

#### 2.4.2 DNA extraction

DNA extraction was performed following the manufacturer’s guidelines using the DNA Mini Kit from Sangon Biotech, Shanghai, China. The concentration of DNA was measured using a nucleic acid protein detection instrument from Thermo Scientific NanoDrop, NanoDrop Technologies, Wilmington, DE, USA. The extracted DNA was deemed suitable for storage at −80°C for future PCR sequencing analysis.

#### 2.4.3 DNA methylation analysis

The EpiTect Bisulfite Sequencing Reagent Kit (Qiagen) was employed for bisulfite sequencing of genomic DNA. Utilizing the Acegen Targeted Methyl Panel multi-target bisulfite sequencing system software, iterative rounds of bisulfite PCR amplification were conducted on the specified target sequence (mitochondrial gene ID: NC_012920.1) utilizing two distinct pools of primers (upstream primer pool A and downstream primer pool B, with primer lengths ranging from 26 to 40 bases and an annealing temperature of 55°C–65°C; resulting in amplicon lengths of 150–250 bp). The Acegen Targeted Methyl Panel Kit amplification system was utilized to prepare sequencing libraries. The primer sequences can be found in Supplementary Table 1. Initially, genomic DNA (50–200 ng) was processed and converted with the Zymo EZ DNA Methylation Kit (Zymo Research, Irvine, CA, USA), followed by multiple rounds of bisulfite PCR amplification (approximately 25–33 cycles) for targeted amplification. The resulting DNA was subsequently used for DNA library preparation with the Acegen DNA Library Preparation Kit (Acegen, Cat. AG0810) to create dual-indexed libraries. The libraries that had been prepared underwent qualitative analysis and concentration determination utilizing the Qubit 3.0 and Agilent 2100 Bioanalyzer. Libraries that met the required criteria were then subjected to sequencing using the Illumina NGS sequencing system. The sequencing approach employed was paired-end indexed sequencing with a read length of PE150, and a coverage depth of at least 200×. Further information on the specific procedures can be found in the provided reference (Wang et al., 2023).

#### 2.4.4 Mitochondrial copy number

In this investigation, the relatively stable expressed genes Hemoglobin β-globin, and MT-ND (Mitochondrially Encoded NADH: Ubiquinone Oxidoreductase Core Subunit 1) were chosen from the human genome and mitochondrial genome for the purpose of mitochondrial copy number determination. A quantity of 20 ng of cellular DNA was utilized as the starting material for quantitative PCR (qPCR). The qPCR reactions were carried out utilizing the StepOnePlus Real-Time System (Applied Biology) with the following parameters: initial denaturation at 95°C for 10 s, denaturation at 95°C for 5 s, annealing at 56°C for 34 s, for a total of 35 cycles. The total volume of the qPCR reaction was 20 μL, consisting of 10 μL of master mix (Tiangen Biochemical Technology, Cat. FP215), 10 pmol of each primer, and 2 μL (20 ng) of DNA. The Ct values were determined using the Applied Biology Manager. The primer sequences for Hemoglobin β (reference gene sequence number: MH708880.1) were as follows: forward primer GCT​TCT​GAC​ACA​ACT​GTG​TTC​ACT​AGC and reverse primer CAC​CAA​CTT​CAT​CCA​CGT​TCA​CC. For the mitochondrial-encoded chrM: 3313-3322 (reference gene sequence number: NC_012920.1), the primer sequences were as follows: forward primer CAC​CCA​AGA​ACA​GGG​TTT​GT and reverse primer TGG​CCA​TGG​GTA​TGT​TGT​TA. The two sets of primers underwent separate amplification processes, with amplification plots subsequently generated using the StepOnePlus Real-Time PCR System. The relative content of MT-ND1 was determined utilizing the CT difference method outlined in existing literature, and the results were expressed as a ratio (Stoccoro et al., 2021).

### 2.5 Analysis

#### 2.5.1 Bioinformatics analysis

High-quality data is essential for accurate methylation analysis, and rigorous quality control is a critical component of the process. Raw sequencing reads were first processed using Trimmomatic v0.36 to remove adapter sequences and low-quality bases. Clean reads were then aligned to the reference mitochondrial genome (NC_012920.1) using BSMAP v2.73 for downstream methylation analysis. To ensure data reliability, a quality threshold of Q20 > 95% was applied, indicating that over 95% of bases had a sequencing accuracy of ≥99% (Lin et al., 2023). This is a widely accepted standard in bisulfite sequencing studies to minimize technical noise (Gong et al., 2022). In this study, the average original Q20 was 96.45%, clean Q20 was 97.62%, and the mapping rate reached 96.45%, all exceeding the 95% threshold and supporting the high quality of the sequencing data. Determine the methylation level of C sites by applying the formula: methylation level of C site = number of reads supporting methylation/(number of reads supporting methylation + number of reads supporting unmethylation). Calculate the methylation ratio of detectable sites in each sample, with a representation of NA for depths below 30X. Functional and differentially methylated gene sites’ signaling pathways can be enhanced through the utilization of the KEGG database with a corrected P-value <0.05 as determined by KOBAS v3.0 (http://bioinfo.org/kobas/).

#### 2.5.2 Statistical analysis

All statistical analyses were conducted using SPSS software (version 16.0, SPSS Inc., Chicago, IL, USA) or R software (version 4.3.2, R Foundation for Statistical Computing, Vienna, Austria). Age and gender distribution within the sample population were compared between groups using t-tests or Chi-square test. The Mann-Whitney U test was employed to validate disparities in mtDNA methylation levels across groups. Spearman’s correlation coefficient was used to calculate the correlation between mtDNA methylation levels and mitochondrial copy number. Two-sided P-values <0.05 were considered statistically significant.

Spearman’s correlation coefficient was utilized to assess the relationship between mtDNA methylation levels and mitochondrial copy number. Statistical significance was determined by two-sided P-values <0.05.

Based on statistical outcomes and literature evidence, key genes exhibiting significant differential methylation were identified. Adjusting for sex as a covariate, logistic regression analysis was performed for each CpG site within these genes. A classifier was then developed employing a multivariate single-gene logistic regression model. The classifier’s performance was evaluated by computing the Receiver Operating Characteristic (ROC) curve and the Area Under the ROC Curve (AUC). Robustness was further validated through five-fold cross-validation. All statistical analyses were conducted using the R software environment (version 4.3.2).

## 3 Result

### 3.1 Basic characteristics of all subjects

A total of 80 participants were included in the study, consisting of 40 individuals with noise-induced hearing loss (15 females, 25 males, mean age of 42.4 ± 5.5 years) and 40 controls (26 females, 14 males, mean age of 42.3 ± 5.6 years). Gender distribution between the two groups showed a statistically significant difference (Chi-square test, Chi = 6.054, p = 0.014), while no significant difference was observed in age (t-test, T = 0.040, p = 0.968). The observed gender imbalance in the NIHL group aligns with previous studies indicating that men are more likely to experience hearing loss, which may be due to higher exposure to occupational and recreational noise. Additionally, anatomical differences such as a longer cochlear length in males (∼1.11 mm) may affect cochlear mechanics and contribute to increased vulnerability to noise-induced damage. Variations in cochlear length are hypothesized to influence the stiffness of the basilar membrane, potentially altering its susceptibility to injury (Bowman et al., 2000).

### 3.2 KEGG PATHWAY functional enrichment analysis

**FIGURE 1:**
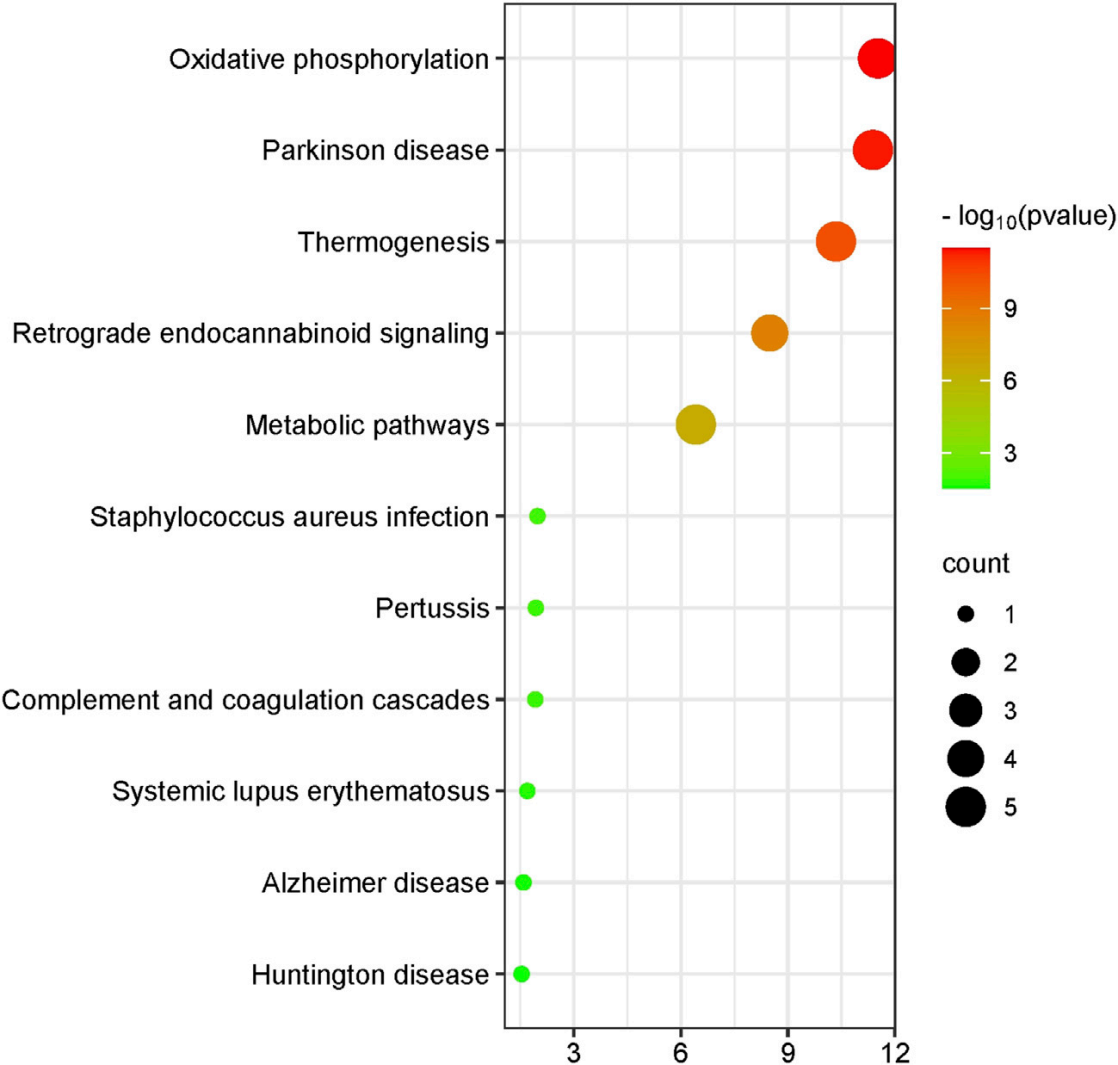
Mitochondrial differential gene visualization enrichment plot. This figure illustrates the results of enrichment analysis for various pathways, with significance levels represented by −log_10_ (*p*-value) and the number of genes indicated by bubble size. The x-axis shows the −log_10_ (*p*-value), while the y-axis lists the pathway names. The color and size of the bubbles represent the significance level and the number of enriched genes, respectively. The color gradient from green to red indicates increasing −log_10_ (*p*-value), and the bubble size indicates the count of enriched genes.

Functional analysis indicated that the differentially expressed genes within the mitochondria are primarily linked to oxidative phosphorylation, Parkinson’s disease, thermogenesis, retrograde endocannabinoid signaling, metabolic pathways, *Staphylococcus aureus* infection, pertussis, complement and coagulation cascades, systemic lupus erythematosus, Alzheimer’s disease, and Huntington’s disease (Figure 1).

### 3.3 Expression levels of antioxidant markers

**FIGURE 2:**
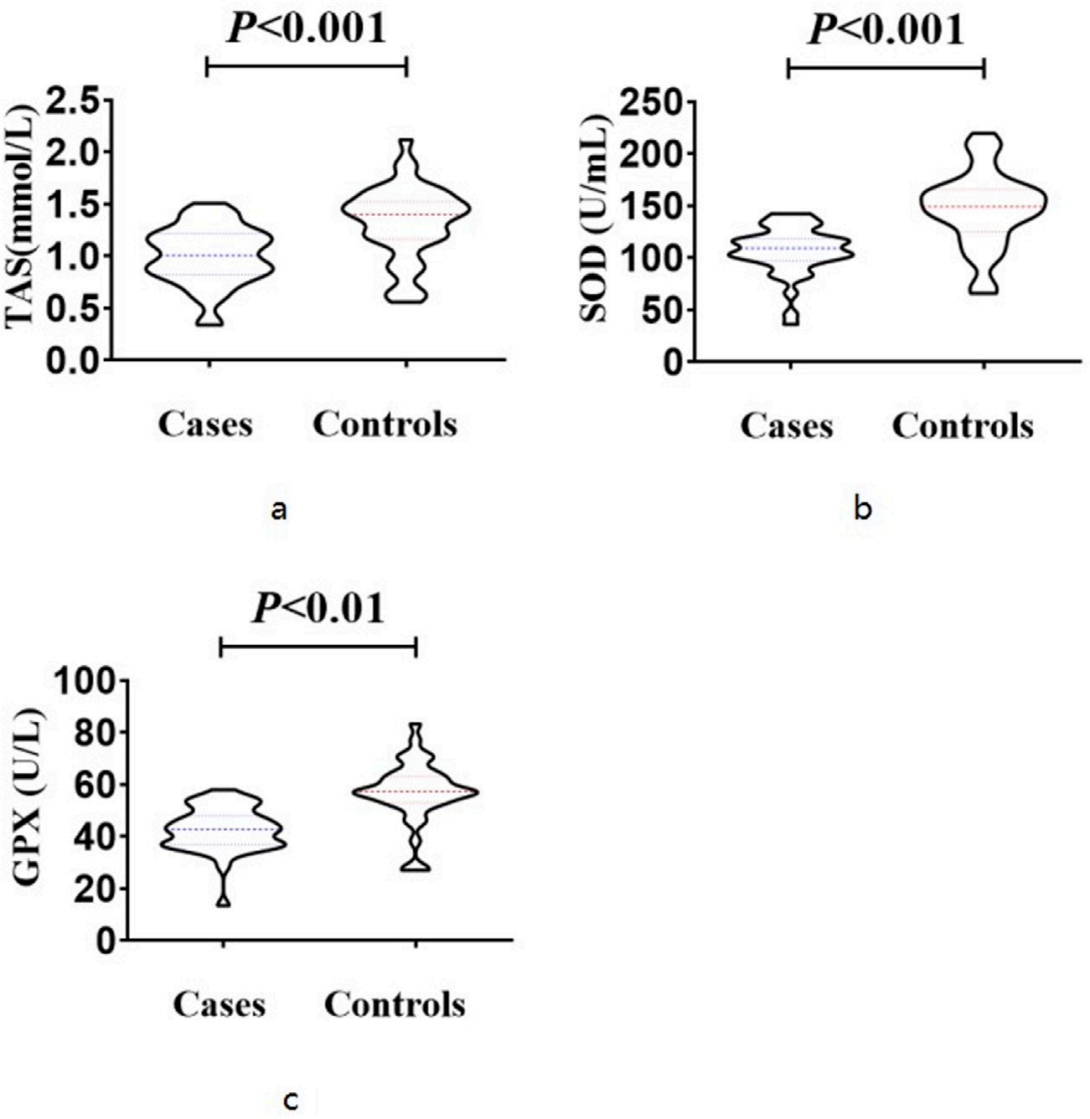
Expression levels of antioxidant markers The case group exhibited significantly lower total antioxidant status (1.008 ± 0.277 mmol/L) compared to the control group (1.325 ± 0.355 mmol/L), as determined by a t-test (T = 4.445, *p* < 0.001) **(a)**. The superoxide dismutase level in the case group (107.1 ± 23.2 U/mL) was significantly lower than that in the control group (147.0 ± 36.3 U/mL) based on a t-test (T = 5.866, *p* < 0.001) **(b)**. The glutathione peroxidase level in the case group (42.80 ± 8.54 U/L) was significantly lower than that in the control group (56.95 ± 11.92 U/L), as indicated by a t-test (T = 2.905, *p* = 0.004) **(c)**.

The case group exhibited significantly lower total antioxidant status (1.008 ± 0.277 mmol/L) compared to the control group (1.325 ± 0.355 mmol/L), as determined by a t-test (T = 4.445, p < 0.001). Similarly, the superoxide dismutase level in the case group (107.1 ± 23.2 U/mL) was significantly lower than that in the control group (147.0 ± 36.3 U/mL) based on a t-test (T = 5.866, p < 0.001). Additionally, the glutathione peroxidase level in the case group (42.80 ± 8.54 U/L) was significantly lower than that in the control group (56.95 ± 11.92 U/L), as indicated by a t-test (T = 2.905, p = 0.004) (Figure 2). These findings suggest a compromised antioxidant defense system in NIHL patients, supporting the role of oxidative stress as a contributing factor in cochlear injury. Reduced levels of SOD, GPX, and TAS may indicate impaired cellular response to reactive oxygen species generated by prolonged noise exposure.

### 3.4 The methylation levels of individual sites

**FIGURE 3:**
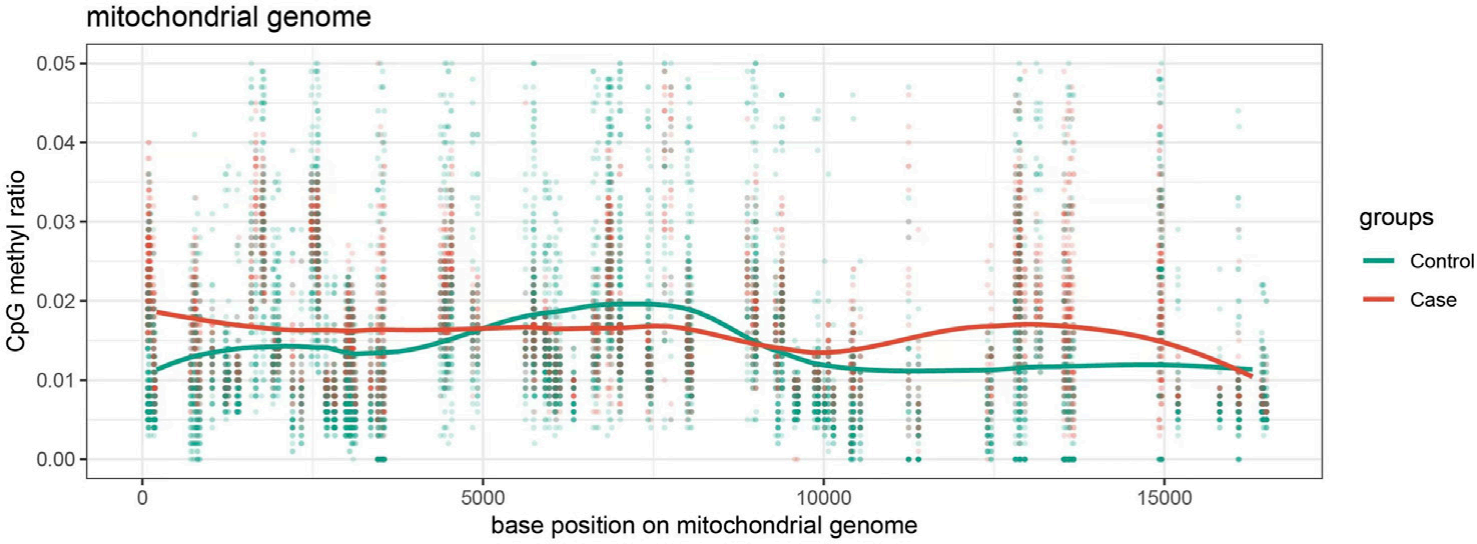
Methylation Level Visualization Line Chart distribution of methylation levels at various loci of mitochondria in the case group and control group, with red representing the case group and green representing the control group.

A comparative analysis was conducted using visualized line charts depicting methylation levels in the two groups. The methylation levels of individual sites within each sample were plotted based on the absolute coordinates of the target gene region. Various colors were used to represent different biological groups, with lines connecting the average methylation levels within each group. The data revealed that the majority of sites in the case group exhibited lower methylation levels in comparison to the control group (Figure 3). These site-specific differences imply that noise exposure may lead to a broad reduction in methylation levels across multiple regions of the mitochondrial genome.

### 3.5 The difference in average methylation levels between the case group and the control group

**FIGURE 4:**
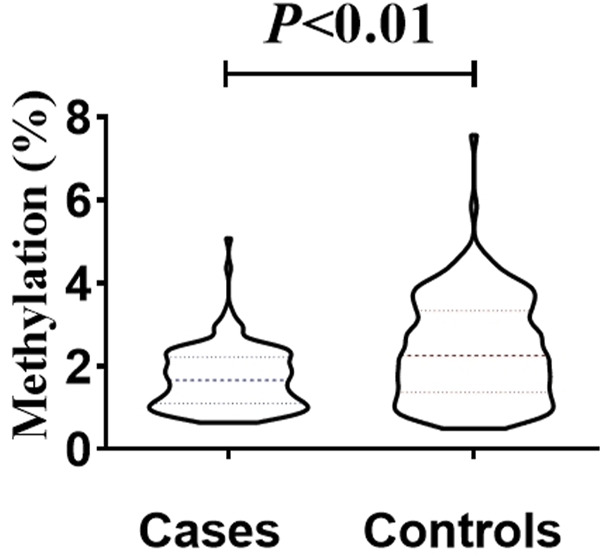
Inter-group differences in overall methylation levels. The overall methylation level in the patient group (median, 1.16%) was lower than that in the control group (median, 2.25%), and the difference was statistically significant (Mann-Whitney test, U = 9454, *p* < 0.01).

The total methylation level in the case group (median, 1.16%) was lower than that in the control group (median, 2.25%), and the difference was statistically significant (Mann-Whitney test, U = 9454, p < 0.01) (Figure 4). This result supports the hypothesis that mtDNA hypomethylation is associated with NIHL and may reflect altered mitochondrial gene regulation in response to chronic oxidative stress.

### 3.6 Disparities in the distribution of methylation sites

**FIGURE 5:**
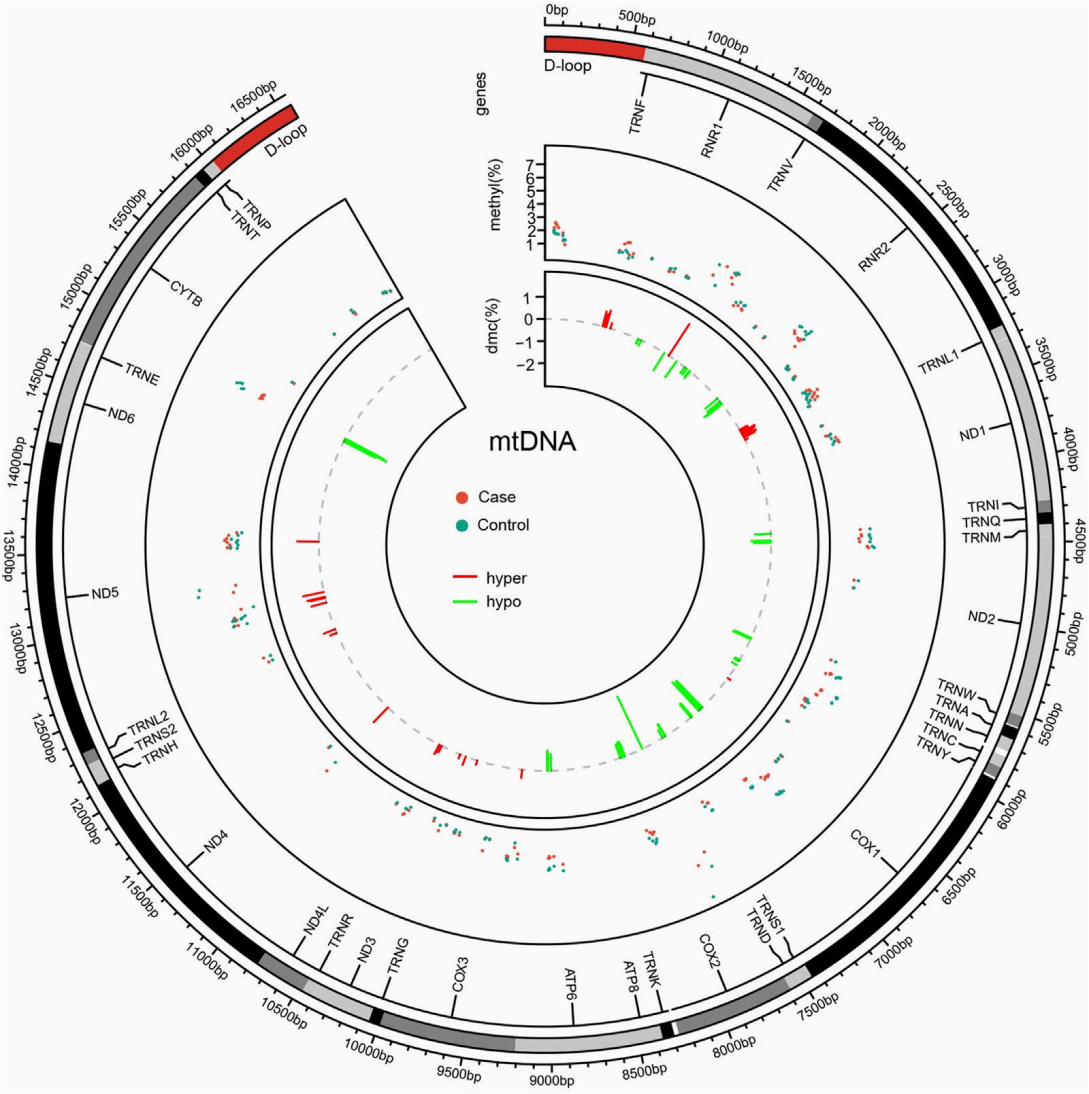
Circos plot of mitochondrial methylation level identified between case and control. Circles from outside to inside indicate the following: (i) mtDNA gene distribution; (ii) distribution of methylation levels at mtDNA detected sites, with red dots representing the case group and green dots representing the control group; and (iii) distribution of differentially methylated sites, between the case and control groups, with the red line representing the elevated level in the case group relative to the control group, and the green line representing the lowered level in the case group compared to the control group.

Disparities in the distribution of methylation sites were observed between the case and control groups, with a total of 168 methylation sites identified. Statistically significant differences in methylation levels were found at 84 sites. Among these, 53 sites showed reduced methylation in the case group (12S_rRNA, 16S_rRNA, tRNA-Ile, ND2, tRNA-Trp, CO1, CO2, ATP6, and CYB), while 31 sites showed increased methylation (12S_rRNA, tRNA-Val, 16S_rRNA, CO1, CO3, ND3, tRNA-Arg, ND4, and ND5). Interestingly, the ATP6 and CYB genes exhibited consistent trends that mirrored the group-wide differences, highlighting their potential relevance as biomarkers or mechanistic contributors in NIHL (Table 1; Figure 5).

**TABLE 1:** Comparison of ATP6 and CYB methylation levels in the case and control groups.

	Case (median, %)	Control (median, %)	U	*p* value
ATP6 (all)	1.90	2.30	15,746	0.002^*^
ATP6 (male)	1.90	1.90	4303	0.848
ATP6 (female)	1.90	2.65	3231	<0.001^*^
CYB(all)	2.00	3.05	14,083	<0.001^*^
CYB(male)	2.20	2.90	3540	0.026^*^
CYB(female)	1.90	3.25	2995	<0.001^*^

Note: U: Mann-Whitney U test,*p < 0.05.

### 3.7 Mitochondrial copy number

**FIGURE 6:**
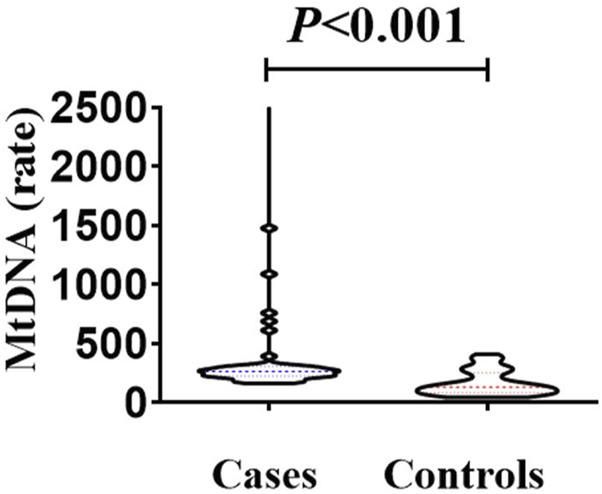
Comparison of mitochondrial gene content between the case group and the control group. The median mitochondrial gene content in the case group (265.1, IQR 226.4 ratio) was higher than that in the control group (median 132.5, IQR 84.4 ratio). There was a statistically significant difference in the comparison between the two groups (Mann-Whitney *U* test, *U* = 311.5, *p* < 0.001).

The median mitochondrial gene content in the case group (265.1, IQR 226.4) was significantly higher than that in the control group (median 132.5, IQR 84.4) (Mann-Whitney U test, U = 311.5, p < 0.001) (Figure 6). The elevation in mtDNA copy number may represent a compensatory mechanism in response to mitochondrial dysfunction caused by oxidative stress. It has been hypothesized that increasing the number of mtDNA copies helps maintain mitochondrial membrane integrity and energy production under stress conditions. This response may serve a protective role in preserving cochlear function, particularly under noise- or drug-induced mitochondrial damage (Mei et al., 2020).

### 3.8 Differential methylation analysis

Table 2 displays AUC, Sensitivity, and Specificity for various loci within the ATP6 and CYB gene regions. The findings suggest that the methylation sites within the CYB gene demonstrate substantial AUC values for predicting noise-induced hearing loss (NIHL), notably at positions 14,945 and 14,963, where the AUC values are 0.769 and 0.777, respectively. These loci exhibit significant predictive potential, with Sensitivity values of 0.825 and 0.900, and Specificity values of 0.650 and 0.625, respectively. The methylation sites within the ATP6 gene demonstrate significant predictive capability, particularly at position 9002, where AUC is 0.720. This position exhibits the highest sensitivity (1.000), although the specificity is comparatively low (0.450).

**TABLE 2:** Comparison of AUC, sensitivity, and specificity for different loci of ATP6 and CYB genes.

Gene region (position)	Locus	AUC	Sensitivity	Specificity
ATP6 (8573-9207)	8959	0.707	0.975	0.400
	8998	0.714	0.600	0.800
	9002	0.720	1.000	0.450
	9010	0.715	0.950	0.450
CYB (14,747-15887)	14,921	0.710	0.550	0.825
	14,930	0.738	0.625	0.775
	14,945	0.769	0.825	0.650
	14,958	0.749	0.825	0.600
	14,963	0.777	0.900	0.625

**FIGURE 7:**
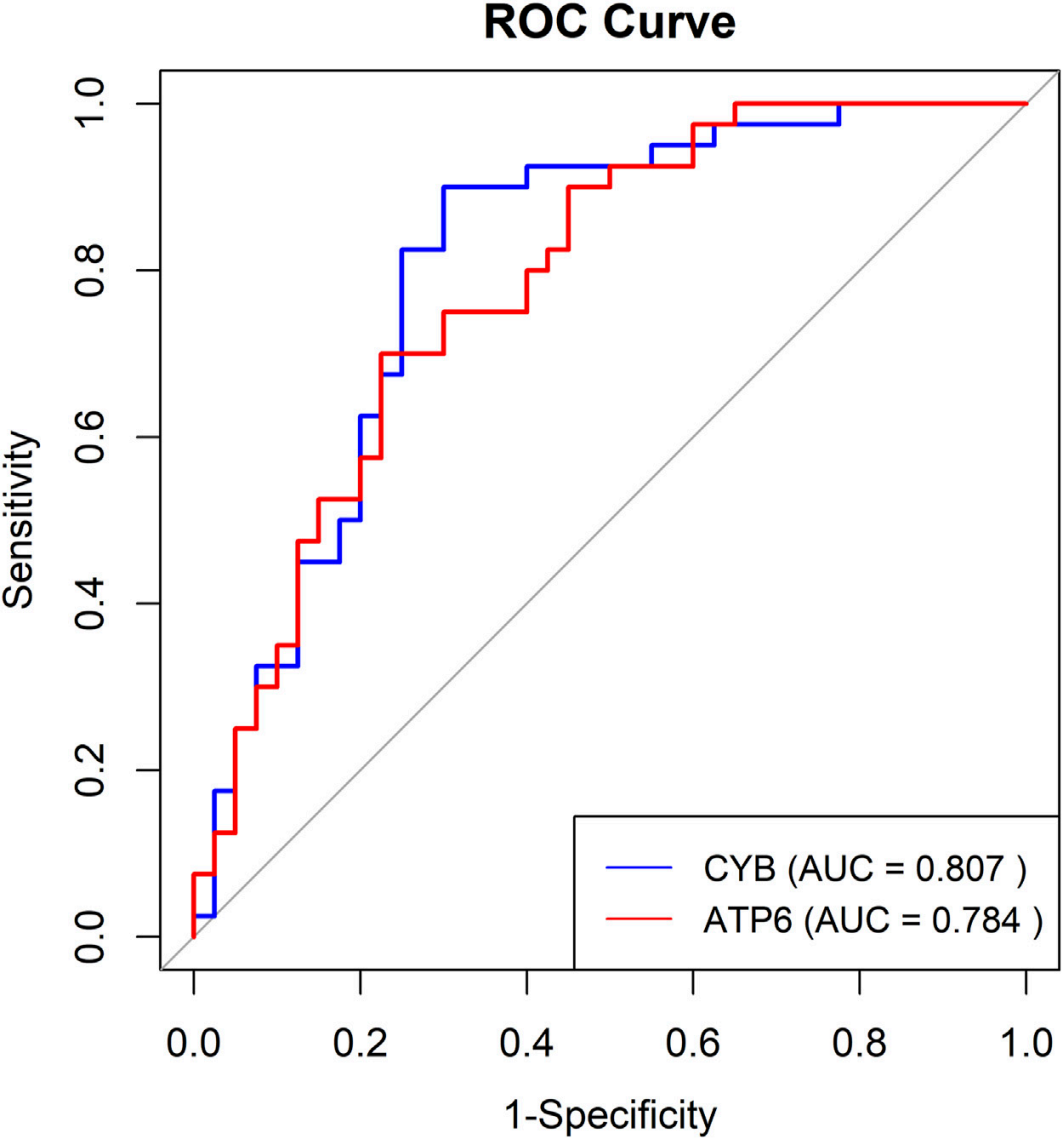
The ROC curves for the multi-locus logistic regression model of ATP6 and CYB genes.

To assess the effectiveness of ATP6 and CYB gene methylation levels in differentiating between individuals with normal hearing and those with NIHL, we conducted logistic regression analysis and constructed the ROC curves, incorporating gender as a covariate in the model (Figure 7). The results indicated that AUC for the CYB gene was 0.807, exhibiting high sensitivity (0.90) and specificity (0.70). Similarly, the AUC for the ATP6 gene was 0.784, with sensitivity and specificity values of 0.70 and 0.775, respectively, suggesting robust predictive performance. Additionally, five-fold cross-validation results revealed AUCs of 0.725 for CYB and 0.734 for ATP6, thereby demonstrating consistent predictive performance across various datasets.

## 4 Discussion

The proliferation of industrial construction has resulted in a corresponding increase in the utilization of various mechanical equipment. Consequently, there has been a rise in the incidence of workplace noise levels surpassing established standards, thereby heightening noise as a prevalent occupational hazard. Exposure to occupational noise has been linked to the development of profound and irreversible hearing impairment, ultimately culminating in deafness. Numerous scholars have endeavored to investigate the pathophysiology of noise-induced hearing loss and furnish empirical data aimed at ameliorating associated auditory deficits (Kurabi et al., 2017). In recent years, there has been a growing interest in the examination of epigenetic modifications, specifically DNA methylation and microRNA profiles, in the context of cochlear nerve cell development and auditory disorders. Yingzi He et al. have demonstrated that the inhibition of DNA methylation via the LRP1-PI3K/AKT (Low-Density Lipoprotein Receptor-Related Protein 1 - Phosphoinositide 3-Kinase/Protein Kinase B) pathway can mitigate oxidative stress-induced mitochondrial-dependent apoptosis and ameliorate cisplatin-induced hearing impairment (He et al., 2022). Yu Zhang and colleagues discovered that children with elevated levels of lead exposure exhibit increased levels of blood DNA methylation in the genes Rb1, CASP8, and MeCP2, as well as higher bilateral average hearing thresholds. The exposed group demonstrated heightened levels of promoter methylation at cg02978827 and position +14, and decreased levels at position +4 of the Rb1 gene (Xu et al., 2020). DNA methyltransferases play a crucial role in catalyzing DNA methylation reactions, which are involved in a diverse array of biological processes and metabolic pathways within the human body, such as gene regulation, cellular differentiation, and tumorigenesis. In the realm of auditory research, existing literature suggests that DNA methylation is implicated in the differentiation and regeneration of auditory hair cells (Liu et al., 2024). Nevertheless, there is a paucity of studies examining the methylation patterns of mitochondrial DNA in individuals afflicted with noise-induced hearing loss. This study determined that cases with noise-induced hearing loss exhibited a decreased level of mitochondrial DNA methylation compared to a control group without hearing impairment. A significant factor in the detrimental effects of noise on hearing is the induction of mitochondrial oxidative stress and the subsequent overproduction of ROS within the mitochondria (Kim et al., 2019). The impairment of mitochondrial function and the buildup of free radicals, including ROS, are recognized as primary cellular mechanisms underlying the apoptosis of cochlear cells resulting from exposure to noise (Benkafadar et al., 2019). The overproduction of ROS resulting from noise exposure can disturb the redox equilibrium, elevate lipid peroxidation levels, and impede the production of endogenous antioxidants, thereby facilitating the release of cytochrome c within mitochondria and initiating the mitochondrial apoptosis pathway in cochlear hair cells (Liu et al., 2022). This accelerated cellular apoptosis ultimately diminishes the overall energy output of mitochondria, necessitating the upregulation of crucial enzymes involved in mitochondrial energy generation. The findings suggest that a reduction in mitochondrial methylation levels may lead to increased expression of pertinent regulatory genes, as evidenced by significantly lower levels of ATP6 and CYB in comparison to the control group.

Mitochondrial genes ATP6 and CYB encode essential subunits of complexes V and III, respectively, and play critical roles in maintaining ATP production and redox balance in cochlear cells. Genetic evidence links ATP6 dysfunction to auditory deficits. A systematic review further associated MT-ATP6 mutations with early-onset hearing impairment in children, highlighting ATP6 as a candidate for functional epigenetic regulation (Roesch et al., 2022). In NARP syndrome, caused by ATP6 mutations, hearing loss frequently co-occurs with neuropathy and retinitis pigmentosa, underscoring the gene’s importance in auditory physiology (Rabinovich et al., 2024). Similarly, CYB mutations have been implicated in mitochondrial disorders that sometimes manifest with hearing impairment (Ghosh et al., 2020). Mechanistic studies in animal models support a role for these mitochondrial genes in noise-induced auditory damage. Acoustic trauma in rats leads to reduced mtDNA content, downregulated mitochondrial gene expression, and decreased ATP levels in cochlear tissue, suggesting functional suppression of genes such as ATP6 and CYB after noise exposure (Chen et al., 2022). Concurrently, noise triggers mitophagy markers (PINK1, Parkin, LC3-II) in the cochlea, indicating a cellular response to mitochondrial damage that may involve altered expression or turnover of mtDNA-encoded subunits (Li et al., 2023). Comprehensive reviews emphasize that mitochondrial dysfunction—characterized by diminished ATP synthesis and elevated ROS—is central to NIHL pathogenesis; given ATP6’s and CYB’s roles in complexes V and III, respectively, epigenetic regulation of these genes is biologically plausible and merits further study (Mao and Chen, 2021; Zou et al., 2022). Consistent with epigenetic regulation of mitochondrial genomes reported in bovine oocyte, hypermethylation of ATP6 and CYB may suppress electron transport chain activity (Sirard, 2019). Public RNA-seq data (GSE52415) confirms an inverse methylation-expression relationship for these genes, supporting their functional role in metabolic reprogramming. Xiaoyang Dou compared the associations between methylation and transcript abundance within each sample and observed a negative correlation between gene body methylation on both strands of mtDNA and transcript abundance (Dou et al., 2019). These studies have all demonstrated the negative regulatory effect of mitochondrial methylation on its target genes.

Our pathway enrichment analysis revealed significant overlap with mitochondrial-related pathways (e.g., oxidative phosphorylation, Parkinson’s disease, thermogenesis) and immune/inflammatory processes (e.g., complement activation, systemic lupus erythematosus), all implicated in NIHL pathogenesis. Differential methylation analysis revealed that methylation levels of ATP6 and CYB distinguish NIHL cases from controls with high predictive performance. Specifically, CYB methylation yielded an AUC of 0.807, with sensitivity of 0.90 and specificity of 0.70, indicating strong biomarker potential for early detection of NIHL. These findings underscore the relevance of mitochondrial epigenetic alterations in NIHL and support further functional validation of ATP6 and CYB methylation in cochlear pathology.

Noise-induced cochlear ischemia and hypoxia play a critical role in initiating oxidative stress responses (Wong and Ryan, 2015). Exposure to high-intensity noise leads to a transient increase followed by a sustained reduction in cochlear blood flow, resulting in ischemia-reperfusion injury and excessive production of ROS (Miguel et al., 2018; Teraoka et al., 2024). These ROS mediate cochlear damage through lipid peroxidation, DNA damage, protein denaturation, and mitochondrial dysfunction, ultimately leading to hair cell death (Fetoni et al., 2019; Yang Z. J. et al., 2024). In addition to direct cellular damage, ROS can activate inflammatory signaling pathways, such as NF-κB, promoting the release of pro-inflammatory cytokines (e.g., IL-6, TNF-α) and triggering a systemic inflammatory response (Greif and Eichmann, 2014; Herzog et al., 2019). This systemic response facilitates the diffusion of oxidative stress signals into the peripheral blood, where elevated ROS levels have been observed post-noise exposure. Furthermore, ROS can influence gene expression via epigenetic mechanisms, including histone modifications and DNA methylation (Provenzano and Domann, 2007). Evidence suggests that oxidative stress-induced epigenetic changes in mitochondrial genes (e.g., altered methylation of MnSOD) may contribute to systemic manifestations of noise-induced damage, providing a potential link between cochlear injury and peripheral biomarkers such as mitochondrial DNA methylation in blood cells (Kroller-Schon et al., 2018).

Importantly, the rationale for selecting blood mitochondria as a biomarker lies in the fact that blood samples are relatively easy to obtain, and the primary source of mitochondria in peripheral blood is leukocytes. Other cellular components, such as platelets, contribute minimally to the mitochondrial pool and therefore do not significantly interfere with the main assay results. Moreover, mitochondrial DNA methylation in blood has already been utilized as a biomarker in other diseases (e.g., Alzheimer’s disease), and previous studies have demonstrated that peripheral blood markers can indirectly reflect tissue damage, such as that in the brain (Stoccoro et al., 2017; Stoccoro et al., 2021; Yue et al., 2022; Zhang et al., 2024).

This study conducted a systematic investigation into alterations in mitochondrial whole-genome methylation among a cohort of individuals with noise-induced hearing loss, identifying numerous instances of aberrant methylation in mitochondrial genes. These findings offer novel insights for the early detection and management of noise-induced hearing loss. Mitochondrial DNA copy number serves as a metric for the abundance of mitochondrial genomes and is commonly utilized as a biomarker for mitochondrial oxidative stress and general dysfunction. Previous research has demonstrated a correlation between mitochondrial DNA copy number and occupational hazards exposure with an increase in mitochondrial copy number observed in populations exposed to organic solvents (Yang Y. et al., 2024). The utilization of mitochondrial DNA copy number as a biomarker for mitochondrial function is growing in prevalence, as it serves as an indicator of the level of mtDNA damage. In the present investigation, individuals with noise-induced hearing loss exhibited a notable elevation in mtDNA copy number when compared to the control group. This observed rise in mitochondrial DNA copy number among patients with noise-induced hearing loss may be attributed to a variety of factors. The process of noise-induced apoptosis in target cells leads to an escalation in free radicals and oxidative stress, consequently influencing alterations in mitochondrial biosynthesis. Disruption of mitochondrial dynamics, particularly in the division process, can lead to an incomplete process, resulting in an elevation in mitochondrial quantity and corresponding mtDNA replication number (Sabouny and Shutt, 2021). Additionally, this study demonstrates that individuals with noise-induced hearing loss exhibit a decreased level of mitochondrial methylation compared to a control group. Methylation plays a negative regulatory role in mitochondrial replication, potentially elucidating the observed increase in mitochondrial DNA copy number. GPX and SOD and TAS expression levels are aberrant in noise-induced hearing loss patients indicated an increase in oxidative stress levels (Kil et al., 2007).

The findings of the present study indicate that elevated mitochondrial DNA copy number may serve as a compensatory mechanism to uphold adequate levels of adenosine triphosphate synthesis. Our research revealed also that individuals in the case group exhibited significantly diminished levels of superoxide dismutase, glutathione peroxidase, and total antioxidant status compared to those in the control group. The collaborative function of various antioxidant enzymes is essential to combat the excessive production of ROS, with superoxide dismutase playing a pivotal role in the generation of oxygen free radicals (Baranoski et al., 2024). SOD functions as a protective mechanism against oxidative stress induced by heightened levels of oxygen free radicals, safeguarding vital organs from harm, a finding that aligns with the conclusions drawn from Mengli Zheng’s study (Zheng et al., 2023). Exposure to noise has been shown to diminish SOD levels, leading to the buildup of superoxide radicals (Hahad et al., 2024). The body’s inability to promptly eliminate ROS results in sustained lipid damage within biological membranes, culminating in oxidative stress. Glutathione peroxidase, a key antioxidant enzyme in humans, plays a crucial role in neutralizing lipid peroxides induced by ROS and superoxide anions (Panday et al., 2020). Animal studies have demonstrated the significant role of glutathione peroxidase in mitigating lipid peroxidation, preserving cell membrane integrity and function, and reducing the incidence of cellular mutations (Flohe et al., 2022). Additionally, the research posits that the decline in glutathione peroxidase levels may be attributed to heightened levels of free radicals induced by chemical toxicity, resulting in damage to mitochondria, endoplasmic reticulum, and other cellular organelles, thereby compromising the synthesis of glutathione peroxidase and elevating its utilization (Lapenna, 2023). The total antioxidant status is indicative of the capacity of low-concentration antioxidants to inhibit the oxidation of free radicals efficiently (Silvestrini et al., 2023). This can occur through direct interaction with free radicals or through the consumption of substances that readily produce free radicals, thus preventing subsequent reactions. Kapoor’s research revealed that exposure to noise leads to a reduction in total antioxidant status, potentially as a result of heightened free radical production negating the antioxidant properties (Kapoor et al., 2011). This study revealed a significant decrease in the levels of superoxide dismutase, glutathione peroxidase, and total antioxidant status in the case group compared to the control group, suggesting impairment of the antioxidant system in individuals exposed to noise-induced elevated free radicals.

DNA methylation, often referred to as the fifth base, is capable of modulating gene expression without modifying the DNA base composition, thereby influencing the activity of downstream proteins. The regulatory function of 5 mC begins with its interference in transcription initiation, ultimately resulting in either gene silencing or activation. Ofer Yizhar-Barnea and colleagues conducted an analysis of methylation differential sites in the mouse inner ear sensory epithelium during critical developmental and maturation periods. Utilizing computational simulations of regulatory networks, they identified key regulators, including Atoh1 and Stat3, that are associated with pathways involved in cell lineage determination and maturation, such as the Notch pathway. Additionally, the researchers identified a putative enhancer characterized by a low methylation region (LMR) that enhances the expression of the GJB6 gene and adjacent non-coding RNA (Yizhar-Barnea et al., 2018). Research on the epigenetic mechanisms underlying hearing loss predominantly emphasizes chromosomal gene methylation, with mitochondrial gene methylation being less common and primarily associated with neurodegenerative disorders of the brain. Talisa K Silzer conducted an analysis of methylation patterns in postmortem cerebellar tissue samples from elderly individuals diagnosed with Alzheimer’s disease (AD), progressive supranuclear palsy (PSP), or pathological aging (PA), revealing varying levels of methylation at position 9 within 11 mitochondrial TRNA genes (Silzer et al., 2020). Andrea Stoccoro conducted an assessment of methylation levels within the mtDNA D-loop region in the blood DNA of individuals with Alzheimer’s disease, revealing a notable 25% reduction in DNA methylation levels (Stoccoro et al., 2017). This finding suggests a significant involvement of mtDNA epigenetic alterations in the development of Alzheimer’s disease. Additionally, Yingying Xu utilized pyrosequencing to examine changes in mtDNA methylation within the CYTB and COX II genes in the hippocampus of APP/PS1 transgenic mice with Alzheimer’s disease (Xu et al., 2021). They also observed elevated methylation levels in these genes, along with a concurrent decrease in mtDNA copy number and expression. Olaia Martínez-Iglesias analyzed the methylation status in a transgenic mouse model of AD and found that global methylation and hydroxymethylation levels were decreased (Martinez-Iglesias et al., 2020). Lun Kuo found that accelerated epigenetic aging, relative to physiological aging, is associated with hearing loss (Kuo et al., 2021). In the broader context, our study aligns with findings from neurodegenerative research. For instance, decreased mtDNA methylation has been reported in Alzheimer’s disease models, and genes involved in oxidative phosphorylation are frequently altered. Pathway enrichment analysis in our study linked ATP6 and CYB to neurodegeneration-associated pathways, reinforcing the relevance of mitochondrial epigenetics in NIHL.

However, this study has several limitations. First, the sample size was modest, and demographic imbalance (more males in the case group) could introduce bias. Second, lifestyle and comorbidities—such as diet, exercise, sleep, or inflammatory diseases—were not fully accounted for. Third, environmental exposures like chemicals or pollutants may influence methylation patterns and were not systematically controlled.

Fourth, while we identified differentially methylated sites in mitochondrial genes such as ATP6 and CYB, gene expression data were not available, which limits our ability to confirm the functional consequences of these epigenetic changes. To further explore the causal mechanisms, we recommend future studies employing animal models subjected to controlled noise exposure. Such models would allow precise investigation of mtDNA methylation changes in the cochlea and auditory cortex, ideally combined with transcriptomic or proteomic analyses to evaluate corresponding gene expression levels. Additionally, longitudinal cohort studies of noise-exposed workers could help identify early biomarkers and track epigenetic changes over time. The generalizability of our findings is also limited, as the study focused on the Han Chinese population. Future research should include multi-ethnic and geographically diverse cohorts to assess whether these biomarkers are consistent across populations. Beyond diagnostic applications, mitochondrial epigenetics may inform therapeutic strategies. Emerging research links mitochondrial methylation to drug metabolism, suggesting potential for personalized interventions. Targeting mtDNA methylation through drugs or gene editing may offer new treatments for NIHL and related disorders.

This study demonstrates that noise-induced hearing loss (NIHL) is closely associated with changes in mitochondrial DNA methylation patterns, particularly in the ATP6 and CYB genes. Mitochondria play a crucial role in energy metabolism and cell survival, and alterations in DNA methylation could directly affect the function of auditory cells. Given the central role of oxidative stress and ROS in noise-induced cellular damage, our findings suggest that mitochondrial DNA methylation, especially in the CYB gene, may serve as a potential biomarker for NIHL.

In addition to alterations in gene methylation levels, mitochondrial oxidative stress triggered by noise exposure might also have an impact on drug metabolism and the body’s response to therapeutic interventions (Choi and Choi, 2015). Mitochondrial DNA methylation is likely to be of crucial significance in modulating the expression of drug-metabolizing enzymes, subsequently influencing the effectiveness and toxicity of drugs (Habano et al., 2015; Tang and Chen, 2015). For instance, the application of mitochondrial-targeted therapies has been extensively investigated in a diverse range of diseases, such as cancer, neurodegenerative conditions, and metabolic syndromes (Zinovkin and Zamyatnin, 2019; Klemmensen et al., 2024). Looking ahead, the combination of mitochondrial DNA methylation biomarkers and drug-targeted therapies holds the potential to offer novel perspectives for personalized treatment approaches in the context of NIHL (Leso et al., 2020). Thus, mitochondrial DNA methylation biomarkers not only hold promise for early detection of NIHL but could also serve as new targets for drug development, disease prevention, and personalized treatment strategies.

## 5 Conclusion

Our findings demonstrate that NIHL is closely associated with altered mitochondrial DNA methylation, particularly in ATP6 and CYB. These epigenetic changes may serve as early indicators and potential therapeutic targets for hearing loss. Mitochondrial DNA methylation not only reflects oxidative stress but also holds promise for improving NIHL prevention, diagnosis, and treatment through precision medicine approaches.

## Data Availability

The data analyzed in this study is subject to the following licenses/restrictions: The data generated and analyzed during this study, including individual health and chemical concentration data, are not publicly available due to sensitivity reasons. However, the data can be made available upon reasonable request to the corresponding authors, subject to the establishment of data-sharing agreements. Requests to access these datasets should be directed to Dianpeng Wang, szpcr@126.com.

## Glossary

12S_rRNA: 12S ribosomal RNA
16S_rRNA: 16S ribosomal RNA
ABTS: 2,2-azinobis(3-ethylbenzothiazoline-6-sulfonic acid) diammonium salt
AD: Alzheimer’s Disease
ADP: Adenosine diphosphate
ATP: adenosine triphosphate
ATP6: ATP synthase F0 subunit 6
AUC: the Area Under the Curve
CO1: Cytochrome c oxidase subunit 1
CO2: Cytochrome c oxidase subunit 2
CO3: Cytochrome c oxidase subunit 3
CpG: Cytosine-phosphate-Guanine
CYB: Cytochrome b
EDTA: Ethylenediaminetetraacetic acid
EGTA: Ethylene glycol-bis(2-aminoethylether)-N,N,N′,N′-tetraacetic acid
GPX: Glutathione Peroxidase
KEGG: Kyoto Encyclopedia of Genes and Genomes
LMR: low methylation region
LRP1-PI3K/AKT: Low-Density Lipoprotein Receptor-Related Protein 1-Phosphoinositide 3-Kinase/Protein Kinase B
mtDNA: mitochondrial DNA
MT-ND1: Mitochondrially Encoded NADH: Ubiquinone Oxidoreductase Core Subunit 1
NADPH: Nicotinamide adenine dinucleotide phosphate
ND2: NADH dehydrogenase subunit 2
ND3: NADH dehydrogenase subunit 3
ND4: NADH dehydrogenase subunit 4
ND5: NADH dehydrogenase subunit 5
NGS: Next-Generation Sequencing
PA: pathological aging
PCR: Polymerase Chain Reaction
PSP: Progressive Supranuclear Palsy
qPCR: Quantitative Polymerase Chain Reaction
ROC: Receiver Operating Characteristic
ROS: reactive oxygen species
SOD: Superoxide Dismutase
tRNA-Ile: Transfer RNA Isoleucine
tRNA-Trp: Transfer RNA Tryptophan
tRNA-Val: Transfer RNA Valine
tRNA-Arg: Transfer RNA Arginine
TAS: Total Antioxidant Status
